# Role of Shiga Toxins in Cytotoxicity and Immunomodulatory Effects of *Escherichia coli* O157:H7 during Host-Bacterial Interactions in vitro

**DOI:** 10.3390/toxins12010048

**Published:** 2020-01-14

**Authors:** Andrea Cecilia Bruballa, Carolina Maiumi Shiromizu, Alan Mauro Bernal, Gonzalo Ezequiel Pineda, Florencia Sabbione, Analia Silvina Trevani, Leticia Verónica Bentancor, María Victoria Ramos, Romina Jimena Fernández-Brando, Manuel Javier Muñoz, Marina Sandra Palermo

**Affiliations:** 1 Laboratorio de Patogénesis e Inmunología de Procesos Infecciosos, Instituto de Medicina Experimental (IMEX), Consejo Nacional de Investigaciones Científicas y Técnicas-Academia Nacional de Medicina, Buenos Aires C1425AUM, Argentina; andrea.bruballa@gmail.com (A.C.B.); alanmbernal@gmail.com (A.M.B.); gonzalopineda08191@yahoo.com (G.E.P.); toresani7@hotmail.com (M.V.R.); fernandezbrandoromina@gmail.com (R.J.F.-B.); 2 Laboratorio de Inmunidad Innata, Instituto de Medicina Experimental (IMEX)-CONICET, Academia Nacional de Medicina, Buenos Aires C1425AUM, Argentina; maiumi.shiromizu@gmail.com (C.M.S.); florenciasabbione@hotmail.com (F.S.); analiatrevani@yahoo.com.ar (A.S.T.); 3 Laboratorio de Ingeniería Genética y Biología Celular y Molecular, Universidad Nacional de Quilmes, Buenos Aires 1876, Argentina; lvbentancor@gmail.com; 4 Instituto de Fisiología, Biología Molecular y Neurociencias (IFIBYNE-UBA-CONICET), Departamento de Fisiología, Biología Molecular y Celular and Departamento de Biodiversidad y Biología Experimental, Facultad de Ciencias Exactas y Naturales, Universidad de Buenos Aires, Ciudad Universitaria, Buenos Aires C1428EHA, Argentina; manolo2003@gmail.com

**Keywords:** EHEC, Stx2, *stx_2_*, macrophages, intestinal epithelial cells, IL-1β, IL-8, mRNA

## Abstract

Enterohemorrhagic *Escherichia coli* (EHEC) strains are food-borne pathogens that can cause different clinical conditions. Shiga toxin 2a and/or 2c (Stx2)-producing *E. coli* O157:H7 is the serotype most frequently associated with severe human disease. In this work we analyzed the hypothesis that host cells participate in Stx2 production, cell damage, and inflammation during EHEC infection. With this aim, macrophage-differentiated THP-1 cells and the intestinal epithelial cell line HCT-8 were incubated with *E. coli* O157:H7. A time course analysis of cellular and bacterial survival, Stx2 production, *stx_2_* transcription, and cytokine secretion were analyzed in both human cell lines. We demonstrated that macrophages are able to internalize and kill EHEC. Simultaneously, Stx2 produced by internalized bacteria played a major role in macrophage death. In contrast, HCT-8 cells were completely resistant to EHEC infection. Besides, macrophages and HCT-8 infected cells produce IL-1β and IL-8 inflammatory cytokines, respectively. At the same time, bacterial *stx_2_*-specific transcripts were detected only in macrophages after EHEC infection. The interplay between bacteria and host cells led to Stx production, triggering of inflammatory response and cell damage, all of which could contribute to a severe outcome after EHEC infections.

## 1. Introduction

Enterohemorrhagic *Escherichia coli* (EHEC) strains are food-borne pathogens that can cause different clinical conditions, such as self-limited diarrhea, hemorrhagic colitis, and systemic complications, such as hemolytic-uremic syndrome (HUS) [1,2,3,4]. One of the EHEC strain most frequently associated with severe human disease is *E. coli* O157:H7 [5].

EHEC enters the gastrointestinal tract, survives the acidic condition of the stomach, and reaches intestine, where adhesion to epithelial cells is the first step in the pathogenic cascade. It has been revealed the preferential binding to the follicle associated epithelium (FAE) of Peyer’s patches in the initial events of EHEC colonization, which could lead to the rapid contact of *E. coli* O157:H7 with underlying human macrophages [6]. However, scarce information is available about the interactions between EHEC and these host cells. EHEC O157 from clade 8 carries several virulence factors including Shiga toxin 2a and/or 2c (Stx2), cytolethal distending toxin V (CdtV), EHEC hemolysin (EHEC-Hly), and flagellin [7,8]. The Stx2 is encoded in a lambdoid bacteriophage [9,10], which is an efficient vector for the transfer of *stx* and plays an important role in the evolution of new pathogens [11,12,13]. As a result of prophage induction, host bacteria lyse release Stx2 and free phage particles that can infect other bacteria [14,15,16,17]. However, low levels of spontaneous phage induction can also occur. Transcription of *stx_2_* is highly dependent on induction of the phage lytic cycle, as it is mainly governed by the late phage promoter pR’ [11]. In addition, it has been recently demonstrated that Stx2a and/or Stx2c from periplasmic space could be delivered by outer membrane vesicles (OMVs) [7,18]. A comprehensive understanding of early events during EHEC colonization that lead to HUS could aid in the development of new strategies to prevent and treat the disease.

One way to understand the pathogenesis of HUS is to reproduce host-pathogen interactions on an in vitro model. We have previously reported the ability of eukaryotic cells to recognize putative promoter-like sequences on *stx_2_* driving Stx2 expression by cell lines [19]. Moreover, mouse in vivo transfection with *stx_2_* cloned into a prokaryotic plasmid (pStx2) showed *stx_2_* mRNA in the liver and Stx2 biological toxicity [20]. Therefore, in this work we analyzed the hypothesis that human cell lines participate in Stx2 production after infection with EHEC strains. We first demonstrated that the 293T cell line transfected with pStx2 and transcribed mRNA corresponding to Stx2 A and B subunits, which results in Stx2 biologic activity in the supernatant. Then, we analyzed whether this process could take place in human macrophagic and intestinal epithelial (HCT-8) cell lines during EHEC infection, as an in vitro model closer to the in vivo physiopathologic condition. With this aim, both cellular lines were infected with EHEC O157:H7 isolated from a pediatric HUS patient, and a time course analysis of cellular as well as bacterial survival, Stx2 production, *stx_2_* transcription, and cytokine secretion was done. We found that both cell lines differ markedly in the cellular response to bacterial infection. In fact, we demonstrated that macrophages are able to internalize and kill EHEC. However, HCT-8 cells are not able to eliminate bacteria nor EHEC are able to kill epithelial cells. We analyzed the triggering of inflammatory response and searched eukaryotic *stx_2_* mRNA in both cell types after infection. The interaction between EHEC and human cells could control infection, but also contribute to host damage.

## 2. Results

### 2.1. Expression of Stx_2_ Subunits by 293T Cells

**Figure 1:**
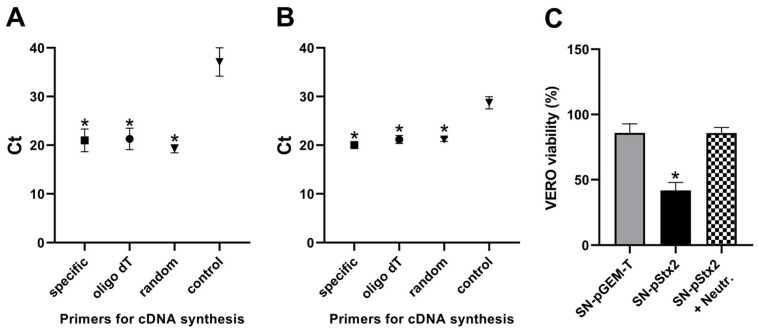
Expression of Stx2-A and -B subunits by 293T cells. 293T cells were transfected with 2 μg of pStx2 or pGEM-T. After overnight (ON) incubation, supernatants were collected (SN-pGEM-T and SN-pStx2) and total RNA from cells was purified. cDNAs were synthesized with the indicated primers. RT-qPCR results for *stx_2_-A* (**A**) or *stx_2_-B* (**B**) are shown as Cycles threshold (Ct) values. cDNA synthesis without reverse transcriptase were used as control (control). (**C**) Stx2 activity in the supernatants was measured by Vero assay. SN-pStx2 was pre-incubated with anti-Stx2 neutralizing antibody (SN-pStx2 + Neutr.). Data represent mean ± SEM for biological replicates (n = 3). (**A**) * *p* < 0.01 and (**B**) * *p* < 0.001, compared with control. (**C**) * *p* < 0.001, compared with SN-pGEM-T and SN-pStx2 + Neutr. (one-way analysis of variance (ANOVA)).

Our first approach was to evaluate *stx_2_* subunits expression by eukaryotic 293T cells after transfection with a prokaryotic plasmid carrying the *stx_2_* sequence (pStx2) or pGEM-T as control. Total RNA was purified and specific transcripts were quantified by RT-qPCR. RNA analysis showed the presence of *stx_2_* mRNA for A (*stx_2_-A*) and B (*stx_2_-B*) subunits. Besides, data revealed similar amounts of transcripts using either oligo (dT), random, or the corresponding subunit-specific primers for cDNA synthesis (Figure 1A,B). Cells transfected with pGEM-T did not show any specific *stx_2_*-transcript (data not shown).

Supernatants (SN) were collected after transfections (SN-pGEM-T and SN-pStx2), and incubated with Vero cells to evaluate Stx2-cytotoxic activity. Only the SN-pStx2 showed cytotoxicity, and significant neutralization of this activity was observed when this supernatant was pre-incubated with an anti-Stx2 neutralizing antibody (Figure 1C). These results suggest that eukaryotic cells can transcribe a plasmid with the *stx_2_* sequence and produce biologically active Stx2 protein, which might be released upon cellular lysis.

The next aim was to analyze whether this process could take place during bacterial-host interactions in the context of in vitro infection of human cell lines with EHEC.

### 2.2. Survival of Human Cell Lines after Infection with EHEC

**Figure 2:**
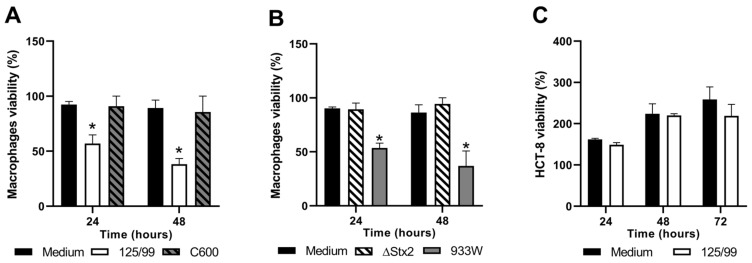
Survival of human cell lines after infection with bacteria. (**A** and **B**) Macrophages were infected with 125/99, 125/99ΔStx2 (ΔStx2), C600, or C600Φ933W (933W) and cell viability was evaluated by MTT assay at 24 and 48 h post-infection with the indicated bacterial strains (n = 4). (**C**) HCT-8 viability was evaluated by MTT assay at 24, 48, and 72 h post-infection with 125/99 (n = 3). Non-infected cells were cultured in medium alone (medium). The absorbance obtained at the beginning of the assay was determined as 100% viability. Data represent mean ± SEM for biological replicates. (**A**) * *p* < 0.001, compared with medium and C600. (**B**) * *p* < 0.0001, compared with medium and 125/99ΔStx2 (two-way ANOVA).

We analyzed the interaction between the pathogenic *E. coli* O157:H7 strain 125/99 and two types of cell lines relevant to the physiopathology of EHEC infections: macrophages and intestinal epithelial cells. The human monocyte cell line THP-1 was differentiated into macrophages with phorbol 12-myristate 13-acetate (PMA) as described in Materials and Methods. These cells were characterized by a significant increase in CD14 membrane expression. Mean fluorescence intensity (MFI) on differentiated THP-1 macrophages was 150% higher than MFI on non-differentiated THP-1 cells, confirming their macrophagic profile (data not shown). Macrophages and 125/99 were coincubated for 20 min at a multiplicity of infection (MOI) 10 as previously reported [21]. After this time, cells were washed and treated with gentamicin (100 μg/mL) to eliminate extracellular bacteria without affecting macrophage viability. Supernatants were collected at different time points, and macrophages were tested by 3-(4,5-DimethylthiaZA-2-yl)-2,5-diphenyltetrazolium bromide (MTT) assay to evaluate the effect of 125/99 infection on cell viability (Figure 2A). Because 125/99 strain expresses a broad spectrum of virulence factors, we assayed in parallel a non-pathogenic *E. coli* strain (C600). As shown in Figure 2A, only the 125/99 strain induced a decrease in macrophage viability by MTT assay, as well as a decline in the number of viable cells measured with the trypan blue staining method (data not shown). To evaluate the role of Stx2 in this detrimental effect on macrophage viability, macrophages were infected with the Stx2-deficient isogenic strain (125/99ΔStx2) or C600 strain carrying the 933W bacteriophage, which expresses Stx2 (C600Φ933W) (Figure 2B). While both Stx2-producing bacteria significantly decreased viability of macrophages, Stx2-nonproducing bacteria (C600 and 125/99ΔStx2) did not affect macrophage viability at any time. These results strongly suggest that Stx2 is responsible for affecting macrophage viability after bacterial infection. The assayed was stopped at 48 h post-infection, because a very low number of macrophages remained with functional capacity.

In sharp contrast, 125/99 strain was not able to kill HCT-8 cells, and they remained viable until 72 h when the assay was stopped (Figure 2C).

### 2.3. Survival of E. coli O157:H7 after Incubation with Human Cell Lines

**Figure 3:**
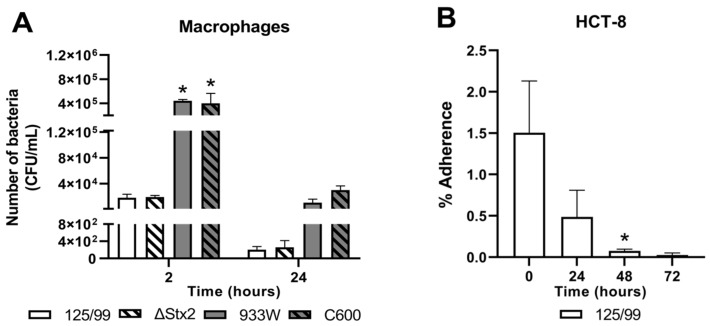
Bacterial survival after incubation with human cell lines. (**A**) The number of bacteria from Triton cell lysates was quantified in macrophages at different times after infection with the indicated bacterial strains. (**B**) Percentage of adherent bacteria to HCT-8 cells was calculated comparing to the number of bacteria added at the beginning of the infection at different times after infection with 125/99. Data represent mean ± SEM for biological replicates (n = 3). (**A**) * *p* < 0.01, compared with 125/99 and 125/99ΔStx2 (ΔStx2) at 2 h post-infection (two-way ANOVA). (**B**) * marginally significant *p* < 0.056, compared with 0 h post-infection (one-way ANOVA).

Bacterial phagocytosis and the fate of intracellular bacteria were determined by CFU quantification in cell lysates. The rate of phagocytosis varied widely between pathogenic and non-pathogenic *E. coli* strains. The number of internalized 125/99 bacteria at 2 h post-infection was significantly lower than C600 in macrophages, suggesting that the ability of macrophages to phagocyte bacteria could be related to their pathogenicity (Figure 3A). On the other hand, the presence of Stx2 did not affect the amount of intracellular bacteria recovered from macrophages (Figure 3A).

No living bacteria were detected in the supernatants of infected macrophages at any time point (data not shown). The number of internalized bacteria decreased during the infection and no living bacteria were recovered at 48 h post-infection, suggesting that all the strains were similarly killed following phagocytosis. Altogether these results indicate that although Stx2 kills macrophages, these cells are able to internalize bacteria and control infection.

On the other hand, a very small number of living bacteria was recovered from HCT-8 cells at all times (at the beginning (time 0), <2% of bacteria added to culture), being almost completely eliminated at 72 h post-infection (Figure 3B). This result is not entirely surprising because HCT-8 cells are not phagocytic, 125/99 are not able to invade them, and also gentamicin (20 μg/mL) was present in the medium during the experiment.

### 2.4. Stx2 Activity in Supernatants from Infected Macrophages and HCT-8 Cells

Considering the differences in cell viability between macrophages and intestinal epithelial cells upon infection with 125/99 bacteria, we further evaluated Stx2 activity in culture supernatants by Vero assay.

**Figure 4:**
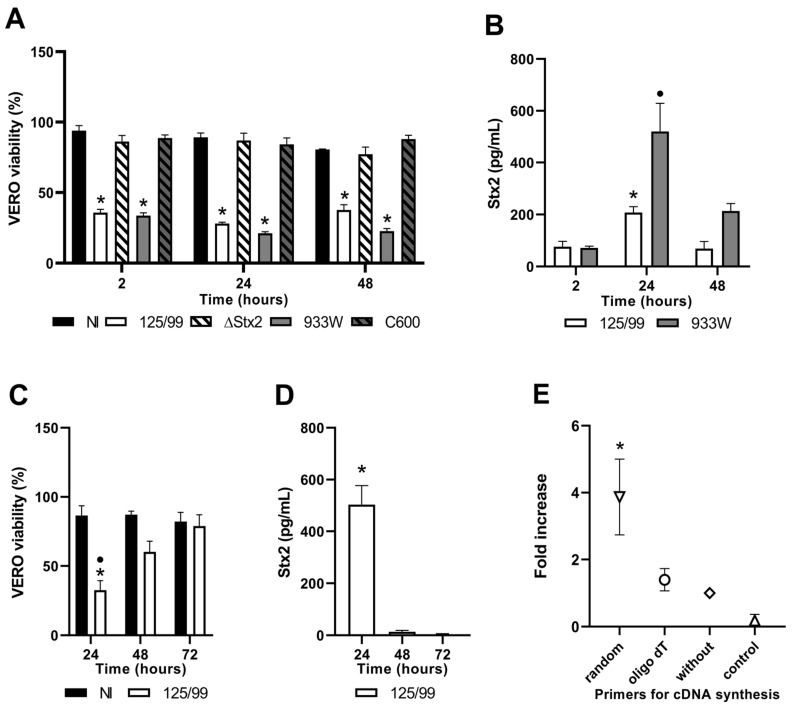
Stx2 activity in supernatants from infected macrophages and HCT-8 cells. Supernatants from macrophages (**A**) or HCT-8 cells (**C**) were obtained at the indicated times after infection with 125/99, 125/99ΔStx2 (ΔStx2), C600 or C600Φ933W (933W), and Stx2-cytotoxic activity was evaluated by Vero assay. Supernatants of non-infected cells were used as controls (NI) and all values were normalized considering Vero cells cultured in medium without any supernatant as 100% viability. The average amount of toxin was calculated in supernatants from macrophages (**B**) or HCT-8 cells (**D**), using a standard curve made with rStx2. (**E**) mRNA quantification for *stx_2_-A* in macrophages 24 h after infection with 125/99 was performed by RT-qPCR with random primers, oligo (dT), or without primer addition. Results are expressed as fold increase, considering RT-qPCR without primers as 1. cDNA synthesis without reverse transcriptase were used as control (control). Data represent mean ± SEM for replicates (n = 3). (**A**) * *p* < 0.0001, compared with NI, C600 and 125/99ΔStx2 (two-way ANOVA). (**B**) * *p* < 0.05, compared with other time points; **•** *p* < 0.001, compared with 2 h post-infection (two-way ANOVA). (**C**) * *p* < 0.05, compared with NI at the same time point; **•** *p* < 0.05, compared with the other time points (two-way ANOVA). (**D**) * *p* < 0.01, compared with the other time points (one-way ANOVA). (**E**) * *p* < 0.05 compared with control (one-way ANOVA).

As depicted in Figure 4A, supernatants from macrophages infected with 125/99 and C600Φ933W strains presented Stx2-specific cytotoxicity, even after 48 h of removal of external bacteria and gentamicin treatment. As expected, supernatants from macrophages infected with C600 or 125/99ΔStx2 did not show cytotoxicity on Vero cells (Figure 4A). To estimate Stx2 concentration in supernatants, we assayed in parallel known concentrations of commercially available recombinant Stx2 (rStx2). For both Stx2-producing strains, the maximal toxin concentration was detected at 24 h (Figure 4B). On the other hand, supernatants from HCT-8 cells infected with 125/99 only showed Stx2-specific cytotoxicity (Figure 4C) and detectable Stx2 concentration at 24 h (Figure 4D). 

Furthermore, lysates from macrophages and HCT-8 cells infected with 125/99 were tested by Vero assay, but not significant Stx2-cytotoxic effect was detected (Figure S1).

### 2.5. Analysis of Stx_2_-gene Expression

Considering that 293T cells transfected with pStx2 expressed mRNA from A and B subunits of Stx2 and the presence of Stx2 in the supernatants from infected cells, we investigated whether macrophages or HCT-8 cells infected with 125/99 transcribed *stx_2_*-mRNA. For this purpose, total RNA was isolated from these cells at 24 h post-infection and *stx_2_-A* was measured by RT-qPCR. In parallel, total RNA was extracted from non-infected macrophages and HCT-8 cells incubated in culture medium as controls. 

*Stx_2_-A* transcripts were only recovered from macrophages infected with 125/99. We used random or oligo (dT) primers for cDNA synthesis to distinguish bacterial from eukaryotic transcripts, considering that eukaryotic mRNA should be amplified whether oligo (dT) is used for cDNA synthesis. S*tx_2_-A* transcripts were only detected using cDNA synthesized with random primers as a template (Figure 4E). These results suggest that Stx2 is produced by internalized bacteria and not by macrophages. As expected, *stx_2_-A* transcripts were not detected in total RNA extracted from non-infected macrophages (data not shown).

On the other hand, *stx_2_-A* transcripts were not detected in total RNA extracted from HCT-8 cells at 24 h post-infection by using random or oligo (dT) primers for cDNA synthesis. These data indicate that HCT-8 cells do not contain *stx_2_-A* mRNA after 125/99 infection, at least in our experimental conditions. 

For both cell lines, *Succinate Dehydrogenase Complex Flavoprotein Subunit A* (*SDHA*) transcripts were detected in total RNA indicating the correct gene expression in eukaryotic cells in our experimental conditions (data not shown).

### 2.6. Mechanisms of Stx2-dependent Cytotoxicity on Infected Macrophages

**Figure 5:**
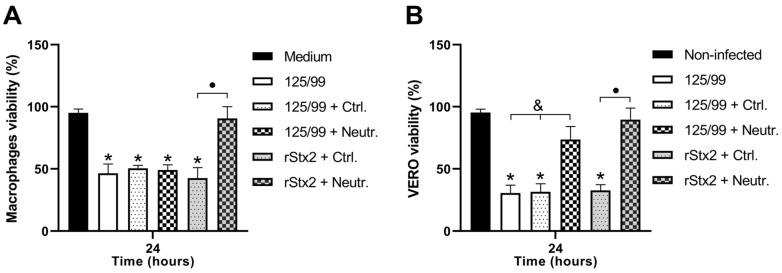
Mechanisms of Stx2-dependent cytotoxicity on infected macrophages. (**A**) Macrophage viability was evaluated by MTT assay at 24 h post-infection with 125/99 incubated in the presence of anti-Stx2 neutralizing antibody (125/99 + Neutr.), non-neutralizing antibody (125/99 + Ctrl.), or without antibody (125/99). Macrophages incubated 24 h with commercial rStx2 (33 pg/mL) and anti-Stx2 neutralizing antibody were used as control of the antibody neutralizing capacity. Non-infected cells were cultured in medium alone (medium). (**B**) Supernatants from macrophages cultured in the conditions indicated in (**A**) were added to Vero cells. Supernatants from non-infected cells were used as control (non-infected) and all values were normalized considering Vero cells cultured in medium without any supernatant as 100% viability. Data represent mean ± SEM for biological replicates (n = 4). (**A**) * *p* < 0.001, compared with medium; • *p* < 0.01, compared with rStx2 + Neutr. (**B**) * *p* < 0.0001, compared with NI; • *p* < 0.001, compared with rStx2 + Neutr.; **&** *p* < 0.01, compared with 125/99 + Neutr. (one-way ANOVA).

We asked whether macrophages were being killed by Stx2 released at the supernatant and subsequent interaction with the membrane Gb3 and/or as consequence of signals triggered by intracellular Stx2. With this aim, an anti-Stx2 neutralizing antibody was added to the culture of macrophages simultaneously with the bacteria. In parallel, macrophages were treated with a non-neutralizing antibody as control. Twenty four hours later, macrophage viability was measured by MTT assay. Results showed that both macrophages incubated in the presence of anti-Stx2 neutralizing or non-neutralizing antibodies reached similar levels of viability (approx. 50%) (Figure 5A). To confirm that the neutralizing antibody was capable of blocking extracellular Stx2, macrophages were incubated in presence of commercial rStx2 (33 pg/mL), together with neutralizing or non-neutralizing antibodies. As can be observed in Figure 5A, the neutralizing antibody completely blocked Stx2-dependent toxicity on macrophages.

Supernatants from each culture condition were tested for Stx2-cytotoxicity by Vero assay. The supernatants from macrophages incubated with commercial rStx2 or 125/99, in both cases in the presence of neutralizing antibody, showed a significantly reduced Stx2 activity (Figure 5B). The fact that specific antibodies to Stx2 did not afford any protection suggests that mechanisms other than those mediated by free Stx2 prevailed in determining macrophage death upon challenge with 125/99.

### 2.7. Intereleukin-1 Betha (IL-1β) Secretion upon Macrophage Infection

**Figure 6:**
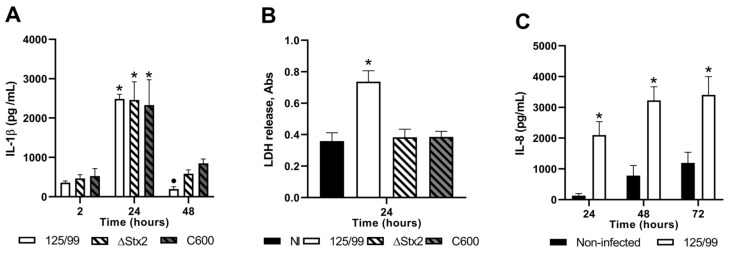
Cytokine secretion upon cell lines infection. (**A**) Concentration of IL-1β in supernatants from macrophages infected with 125/99, 125/99ΔStx2 (ΔStx2), or C600 at different time points (n = 5). (**B**) Lactate dehydrogenase (LDH) released in supernatants from macrophages infected with 125/99, 125/99ΔStx2 (ΔStx2), or C600 or non-infected (NI) (n = 3) at 24 h post-infection. The kit LDH Positive Control showed an absorbance value = 0.735. (**C**) Concentration of IL-8 in supernatants from 125/99 infected or non-infected HCT-8 cells at different time points (n = 3). (**A**) * *p* < 0.01, compared with other time points (two-way ANOVA); • *p* < 0.01, compared with other strains at the same time point (one-way ANOVA). (**B**) * *p* < 0.05, compared with the other strains or NI (one-way ANOVA). (**C**) * *p* < 0.05, compared with non-infected HCT-8 cells at the same time point (two-way ANOVA).

Cytokine secretion by macrophages is one of the major inflammatory responses upon bacterial stimulus. In this regard, TNF-α and IL-1β are the earliest secreted ones with special impact on EHEC pathogenesis [22], because they significantly enhance the toxin-specific glycolipid receptor (Gb3) on the endothelium and Stx2-sensitivity [23]. Thus, we assayed IL-1β levels in the supernatants of macrophages infected with 125/99. It is important to point out that the IL-1β commercial kit only detects 5% of pro-IL1β when both proteins are together (data not shown), suggesting that the values shown correspond mostly to mature IL-1β. Considering that macrophages are exposed to Stx2 as well as other pathogenic factors during bacterial infection, IL-1β was also tested in supernatants from macrophages infected with C600 or 125/99ΔStx2 strains. As depicted in Figure 6A, 125/99, 125/99ΔStx2, and C600 triggered the release of significant amounts of IL-1β at 24 h. Moreover, the IL-1β secretion by macrophages infected with C600 or 125/99ΔStx2 strains was sustained up to 48 h, while IL-1β levels secreted by macrophages infected with 125/99 bacteria decayed at this time point. This different kinetic between Stx2-producing and non-producing bacteria probably reflects the Stx2-dependent cytotoxicity against macrophages, which resulted in a significant decrease of viable cells.

In parallel, lactate dehydrogenase (LDH) release was measured in the supernatants from macrophages at 24 h after infection using a cytotoxicity detection kit. Results showed that the amount of LDH released in the supernatants from macrophages infected with 125/99 was significantly higher than that from macrophages infected with C600 or 125/99ΔStx2 strains, confirming that macrophages infected with 125/99 are dying through a cytotoxic pathway, not preventing IL-1β release (Figure 6B).

### 2.8. Intereleukin-8 (IL-8) Secretion upon HCT-8 Infection

Previous reports have shown that Stx2 treatment of HCT-8 cells elicits significant secretion of IL-8 protein and the related gene expression (mRNA levels) [24]. As depicted in Figure 6C, HCT-8 cells respond to 125/99 incubation with sustained IL-8 secretion over control without bacteria up to 72 h of culture.

## 3. Discussion

While regulation of Stx expression has been extensively investigated in bacterial broth cultures, it remains poorly understood how toxin production is regulated in the complex environment of the human gut. We have previously demonstrated that biologically active Stx2 is produced in vivo after pStx2 transfection, driven by its own *stx_2_* promoter [20]. This evidence raised the question about which is the involvement of host eukaryotic cells in Stx2 production and tissue injury during EHEC infections. Because the first cells to get into contact with EHEC bacteria in the human gut include epithelial cells, macrophages, and dendritic cells, our in vitro approach to simulate the cellular interactions during EHEC infection was the co-culture of a virulent EHEC strain with i) THP-1 derived macrophages or ii) HCT-8 epithelial cells.

In the present work we demonstrated that eukaryotic 293T cells transfected with a pStx2 are able to generate mRNA corresponding to A and B subunits of Stx2, and most importantly, this mRNA has the capacity to translate a biologically active Stx2 protein in the eukaryotic context. Then, we tested the hypothesis that eukaryotic cells participate in *stx_2_* transcription upon interaction with the 125/99 strain. When mRNA was purified from macrophages at 24 h post-infection, we detected *stx_2_-A* transcripts only using cDNA synthesized with random primers as a template by RT-qPCR. These results suggest that Stx2 is produced by internalized bacteria. Since polyadenylation is a characteristic of eukaryotic mRNA, the fact that no *stx_2_-A* transcripts were amplified with cDNA synthesized with oligo (dT) rules out the *stx_2_* transcription by macrophages. When mRNA was purified from HCT-8 cells we could not detect any *stx_2_* signal even when transcripts from the housekeeping gene were identified. In conclusion, after EHEC infection of macrophages or HCT-8 cells we only detected mRNA of bacterial origin within macrophages.

When analyzed biological interaction between macrophages and bacteria, we observed a significant death of macrophages after infection with the 125/99 strain. This effect was time- and Stx2-dependent, but independent of which bacteria produced the Stx2. That is, pathogenic 125/99 or non-pathogenic C600 carrying Stx-phage (C600Φ933W) induced a similar viability loss of macrophages.

Simultaneously, we found that macrophages were able to phagocyte all bacterial strains and kill them. However, we observed a higher number of non-pathogenic C600 compared to 125/99 bacteria within macrophages at 2 h post-infection, independently of Stx2-production, in spite of Poirier et al. [21] showing that both Stx types inhibit EHEC uptake by macrophages. The lower intake of the EHEC strain compared to non-pathogenic bacteria by macrophages suggests that some of the multiple pathogenic factors expressed by EHEC strains, apart from Stx2, modulate phagocytosis by macrophages. This is in agreement with previous reports that have demonstrated that pathogenic bacteria (i.e., EPEC and EHEC) inhibit phagocytosis through several mechanisms, i.e., via inhibition of PI3K activity by proteins codified by the type III secretion system [25,26]. In the same line of evidence, it has been reported that EspF [27], EspB [28], EspJ [29], and EspH [30] inhibit EHEC uptake by macrophages. In spite of the lower intake of pathogenic bacteria, all strains were able to survive within macrophages after 24 h, showing similar numbers of living pathogenic and non-pathogenic bacteria (CFU/mL) in cell lysates at that time. This is in agreement with other authors who reported that macrophages die not only by EHEC infection [31], but also that EHEC can survive and multiply within human macrophages up to 24 h [21,31]. Besides, Stx1/Stx2 is released during this time point [21,32]. In line with these results, the time course analysis of Stx2 in the culture supernatant from infected macrophages showed a significant Stx2 production up to 48 h.

Previous studies revealed that macrophages derived from THP-1 express Gb3 on their membrane [33,34]. As consequence, these cells are able to respond to Stx1/Stx2 [35,36], inducing cytokine/chemokine production, ribotoxic stress, and death of macrophages [22]. Thus, to further elucidate the Stx2-dependent mechanism responsible for macrophage death, we treated cultures with an anti-Stx2 neutralizing antibody since the beginning of 125/99 infection [37]. We observed that Stx2-killing effect on macrophages was not blocked by neutralizing antibodies, although Stx2 activity in these supernatants was specifically and effectively blocked by this antibody, as was demonstrated by Vero assay. In addition, antibody treatment was able to neutralize a CD100% (33 pg/mL) of rStx2 added in the culture medium. These results suggest that other mechanisms than those mediated by free Stx2 prevailed in determining macrophage death upon challenge with 125/99. While several killing mechanisms have been described for cells sensitive to extracellular Stx2, the results described herein led us to hypothesize that killing of macrophages at early times was mediated, at least in part, by intracellular Stx2 produced by bacteria that still remain viable. In this regard, exogenous Stx induces protein synthesis inhibition, but also apoptosis via intrinsic and extrinsic pathways in many cell types [34,38]. However, intracellular Stx2 produced by phagocytized bacteria might be another mechanism for killing macrophages and evading host immune response.

In parallel, supernatants from THP-1 derived macrophages incubated with pathogenic or non-pathogenic strains were tested for inflammatory response by measuring cytokine secretion. Macrophages showed the highest amounts of IL-1β after infection with any bacteria at 24 h. Interestingly, the production of IL-1β was similarly triggered by 125/99, the non-pathogenic C600 bacteria and the 125/99ΔStx2, which expresses all the same pathogenic factors than 125/99 except Stx2. In this sense, it has been previously reported that other pathogenic factors of EHEC strains, such as enterohemolysin encoded in the pO157 virulence plasmid, were involved in IL-1 β secretion [39]. Although IL-1β release is generally associated with death of macrophages, it has been recently demonstrated that bacterial lipopolysaccharides (LPS) induce inflammasome-mediated release of IL-1β from living human cells [40]. In contrast, only the 125/99 strain induced LDH release. LDH and IL-1β levels in supernatants indicated that while infection of macrophages with all strains (125/99, 125/99ΔStx2, or C600) induced IL-1β release, the only one that was associated with a lytic mechanism was 125/99. These results prevent us from ruling out that other lytic mechanisms such as pyroptosis has been involved during 125/99 infection in a Stx2-dependent or independent pathway. Altogether, the simultaneous release of IL-1β and Stx2-dependent cell death triggered by EHEC strain is a very inflammatory condition that could influence the outcome of intestinal infections.

While several previous in vitro studies demonstrated that human macrophages secrete IL-1β following Stx treatment in an NLRP3 inflammasome-dependent manner [35,41,42], scarce reports have analyzed IL-1β secretion following in vitro EHEC infection. In the context of an EHEC infection, Stx2 produced within macrophages could be adding an additional lethal stimulus.

When we similarly analyzed the biological consequences of interaction between HCT-8 cells and bacteria, we found that HCT-8 cells did not diminish viability even after 72 h of infection with 125/99 bacteria. Although this result is not entirely surprising because HCT-8 cells express low levels of Gb3, several authors have reported that they are sensitive to Stx1/Stx2, in a lesser degree compared to Vero cells or human macrophages [43,44]. In parallel, a significant drop in the number of living bacteria since 24 h was observed, in agreement with the fact that they are non-invasive but extracellular living and media-contained gentamicin (20 µg/mL).

Surprisingly, regardless of the different interaction between bacteria and macrophages or HCT-8, we found similar amounts of Stx2 in supernatants from both cell lines, approximately 200–500 pg/mL at 24 h of incubation. These results probably indicate that EHEC-HCT-8 cells interaction leads phage to enter the lytic cycle and release Stx2 to the supernatant. Although we did not rule out that gentamicin triggers Stx2-phage induction, previous reports have also shown that the interaction between the EHEC-Caco-2 cell line induces Stx2 production [32].

On the other hand, we showed that in vitro infection of HCT-8 cells with 125/99 strain triggers IL-8 secretion in line with previous reports [45,46]. Because IL-8 induces recruitment, activation, and migration of neutrophils to the intestine, it could contribute to disruption of epithelial integrity, increasing Stx absorption and thereby the pathogenicity of EHEC [47].

In conclusion, we failed to demonstrate that 125/99-infected human cell lines transcribe the *stx_2_* sequence. However, we could not throw away the hypothesis that the *stx_2_* sequence that may reach host cells by direct bacterial-mammalian interactions as well as by other pathogenic delivery pathways could be processed by eukaryotic translation machine in vivo. In this regard, several recent reports have shown that OMV naturally formed during bacterial infection and released from EHEC can be internalized by eukaryotic cells [48], acting as delivery vehicles for bacterial virulence factors [49,50]. Particularly, it has been demonstrated that OMV from EHEC broth cultures contain Stx2-DNA [7,8,51].

In brief, our results indicate that Stx2 is not involved in the uptake of bacteria by macrophages. Nevertheless, Stx2 produced within macrophages is majorly responsible for IL-1β release associated with a lytic mechanism. During EHEC infection, cells present in the intestine may contribute to pro-inflammatory cytokines release and bacterial production of Stx2. These effects could facilitate EHEC infection and induce intestinal cell damage. Further research in this area may help to develop strategies to interfere with early events in the gut and prevent HUS pathogenesis.

## 4. Materials and Methods

### 4.1. Bacterial Strains

*E. coli* O157:H7 strain 125/99 was isolated from a child with HUS. This strain was from clade 8, harbor *stx_2a_*, but not *stx1* [52,53,54]. The *E. coli* 125/99ΔStx2 strain is a 125/99 isogenic strain that was mutated on Stx2-production and generously provided by Dr. Angel Cataldi et. al. [55]. *E. coli* C600 and *E. coli* C600Φ933W (C600Φ933W) were provided by Dr. Leticia Bentancor et. al. [56]. C600Φ933W is a lysogenized C600 strain carrying the 933W bacteriophage. All strains were cultured overnight (ON) in Tryptic soy broth (TSB) (Difco, Le Point de Claix, France) at 37 °C. ON cultures were diluted 1:100 in RPMI 1640 medium (RPMI) (Gibco, Invitrogen, San Diego, CA, USA) and grown at 37 °C for 2 h. Cultures were centrifuged and bacterial pellets were resuspended in fresh medium. Concentrations of bacteria were determined by measuring absorbance at an optical density of 600 nm.

### 4.2. Cell Lines and Cell Culture

The human monocyte cell line THP-1 (ATCC TIB202) was maintained in RPMI supplemented with 10% heat-inactivated fetal bovine serum (FBS) (Natocor, Córdoba, Argentina), antibiotics (100 U/mL penicillin/streptomycin) (EMEVE Microvet SRL Laboratories, Buenos Aires, Argentina), 0.05 mM β-mercaptoethanol (Sigma, St Louis, MO, USA), and 4 mM L-glutamine (EMEVE Microvet SRL Laboratories, Buenos Aires, Argentina). THP-1 cells were differentiated to macrophages by addition of 10 ng/mL phorbol 12-myristate 13-acetate (PMA) (Sigma, St Louis, MO, USA) for 48 h. To confirm macrophage-like differentiation, PMA-treated and not-treated THP-1 cells were labeled with PECy5-conjugated anti-human CD14 antibody (Clone RMO52) (Beckman Coulter, Brea, CA, USA) or the PECy5 Mouse IgG2a, κ isotype control (Biolegend, San Diego, CA, USA). The fluorescence was measured on 10,000 events by using the Cell Quest program on a FACSCalibur (Beckton Dickinson, San Jose, CA, USA).

Vero cells and the human ileocecal carcinoma cell line HCT-8 (ATCC CCL-244) were grown in RPMI supplemented with 10% heat-inactivated FBS and antibiotics. 293T cells (human embryonic kidney cells) were grown in Eagle’s Minimum Essential Medium (DMEN) (Gibco, Invitrogen, San Diego, CA, USA) supplemented with 10% heat-inactivated FBS and antibiotics. All the cell lines were grown at 37 °C under 5% CO_2_ in a humidified atmosphere.

### 4.3. Infection Assay

THP-1 derived macrophages were seeded at 5 × 10^5^ cells per well in 24-well plates (Greiner Bio-One GmbH, Frickenhausen, Germany) for viability assays and functional studies or 2 × 10^6^ cells per well in 6-well plates (Greiner Bio-One GmbH, Frickenhausen, Germany) for RNA isolation and then bacteria were added to the cell monolayer at a MOI of 10. The plate was centrifuged briefly to synchronize phagocytosis and incubated for 20 min (0 h). Afterward, infected macrophages were washed and fresh medium containing 100 µg/mL of gentamicin (Richet, Buenos Aires, Argentina) was added to kill extracellular bacteria. The supernatants were collected and fresh medium containing 20 µg/mL of gentamicin and 5% heat-inactivated FBS was added after 2 h incubation and every 24 h post-infection. To determine the number of surviving bacteria in the supernatants, pellets obtained by centrifugation were resuspended in phosphate-buffered saline (PBS) and plated onto LB agar. To determine the number of intracellular bacteria in the infected macrophages, a 5 min treatment with 200 µL 0.1% Triton X-100 (Sigma, St Louis, MO, USA) was used to lyse eukaryotic cells at 2, 24, and 48 h. Then, 10-fold dilutions of the lysates were plated onto LB agar and the number of bacteria was determined by CFU. In parallel, to evaluate the Stx2-cytotoxic activity inside the infected macrophages, they were harvested with 400 µL PBS and lysed by four freeze-thaw cycles.

To neutralize the cytotoxic effect of extracellular Stx2 on the infected macrophages, medium supplemented with anti-Stx2 neutralizing antibody or non-neutralizing antibody to a final concentration of 10 nM was added [37]. For RNA extraction macrophages from two different wells were combined and lysed in TRIzol (Invitrogen Life Technologies, San Diego, CA, USA) at 24 h post-infection. RNA from non-infected macrophages was isolated as a control.

### 4.4. Adhesion Assay

HCT-8 cells were cultivated at 2.5 × 10^5^ cells/well in 24-well plates for viability assays and functional studies or 2 × 10^6^ cells per well in 6-well plates for RNA isolation. Cells were washed with PBS, then bacteria were added at an MOI 10 and the culture plate was centrifuged briefly to synchronize adhesion. After incubation for 3 h at 37 °C, non-adherent bacteria were removed by five washes with PBS and fresh medium containing 20 µg/mL of gentamicin was added (0 h). Subsequently, the supernatants were collected and fresh medium containing 20 µg/mL of gentamicin was renewed every 24 h. For quantitative bacterial adherence assays, cell monolayer was gently scraped off with 200 µL 1X-Triton, and the number of adhered bacteria was determined by CFU as described before. In parallel, to evaluate the Stx2-cytotoxic activity inside infected HCT-8 cells, they were harvested with 400 µL PBS and lysed by four freeze-thaw cycles. For RNA extraction, HCT-8 cells from two different wells were combined and lysed in TRIzol at 24 h post-infection. RNA from non-infected HCT-8 cells was isolated as control.

### 4.5. Stx2- cytotoxic Activity on Vero Cells

The supernatants or lysates collected, at the mentioned time points, from the different cell lines (macrophages, HCT-8 and 293T cells) were tested for Stx2-cytotoxic activity on Vero cells as previously described [57]. Briefly, Vero cells were grown in RPMI supplemented with 10% heat-inactivated FBS and antibiotics in 96-wellplates. One in two dilutions of lysates and supernatants were added to each well containing 2 × 10^4^ Vero cells. Cells were incubated at 37 °C in 5% CO_2_ for 48 h. Cells were washed, stained with crystal violet dye, and read on a Microwell System reader 230S (Organon, Teknika, OR, USA) with a 570 nm filter.

The specificity of cytotoxicity of supernatants derived from 293T cells transfected with pStx2 (SN-pStx2) was evaluated in parallel by pre-incubating these samples with an anti-Stx2 neutralizing antibody for 1 h at 37 °C and for 1 h at 4 °C [37]. Known concentrations of rStx2 (Phoenix Lab, Tufts University, Boston, MA, USA) were used to estimate the Stx2 concentration.

### 4.6. Cell Viability Assay

Viability of macrophages and HCT-8 cells was examined using a 3-(4,5-DimethylthiaZA-2-yl)-2,5-diphenyltetrazolium bromide (MTT) (Sigma, St Louis, MO, USA) assay as previously described [58]. Briefly, RPMI with 1:10 volume of MTT solution (5 mg/mL) was added to cell monolayers and incubated at 37 °C for 4 h. The reaction was stopped with acid-isopropanol (100 µL of 0.04 N HCI in isopropanol) and mixed thoroughly to dissolve the formazan crystals. After a few minutes at room temperature to ensure that all crystals were dissolved, the solution was transferred to 96-well plates (Greiner Bio-One GmbH, Frickenhausen, Germany) and analyzed by measuring the absorbance on a Microwell System reader 230S (Organon, Teknika, OR, USA) at 540 and 720 nm. The 720 nm absorbance value (background) was subtracted from the 540 nm absorbance to get a more exact measurement.

### 4.7. Cytokine Assay

The supernatants of macrophages were collected at 2, 24, and 48 h post-infection and the concentration of human IL-1β was quantified by ELISA (BD Biosciences, Franklin Lakes, NJ, USA) following the manufacturer’s instructions and read on a Microwell System reader 230S (Organon, Teknika, OR, USA) with a 450 nm filter. Supernatants from PMA-differentiated THP-1 cells cultured in medium alone served as control for the spontaneous release of cytokine.

The supernatants of HCT-8 cells were collected at 24, 48, and 72 h post-infection and the levels of human IL-8 was quantified by ELISA (BioLegend, San Diego, CA, USA) following the manufacturer’s instructions and read on a Microwell System reader 230S (Organon, Teknika, OR, USA) with a 450 and 570 nm filter. The 570 nm absorbance value (background) was subtracted from the 450 nm absorbance. Supernatants from HCT-8 cells cultured in medium alone served as a control for the spontaneous release of cytokine.

### 4.8. Lactate Dehydrogenase (LDH) Assay

LDH released in the supernatants from macrophages was detected using a cytotoxicity detection kit (Pierce LDH Cytotoxicity Assay Kit), purchased from Thermo Fisher Scientific (Waltham, MA, USA). Data was expressed as Absorbance units (AU).

### 4.9. Plasmid Construction

The plasmid was constructed by standard cloning techniques, according to the NIH policy manual on Working Safely with Hazardous Biological Materials. The complete *stx_2a_* sequence was amplified by PCR from total DNA from *E. coli* C600Φ933W, using the primers *stx_2_*Fw (5′-GAATTCATTATGCGTTGTTAG-3′) and *stx_2_*R (5′-GAATTCTCAGTCATTATTAAACTG-3′), both containing an EcoRI restriction site [18]. The resulting fragment (1413 bp) was cloned in a pGEM-T Easy vector (Promega Inc., Madison, WI, USA), generating the plasmid pStx2. This plasmid and pGEM-T religated vector were replicated in *E. coli* DH5α competent cells in parallel and purified using the Wizard Plus Minipreps DNA purification system (Promega Inc., Madison, WI, USA) following standardized instructions.

### 4.10. Transfection Assay

293T cells were seeded at 6 × 10^5^ cells/well in six-well culture plates. Cells were transfected with Polyfect reagent (QIAGEN Inc, Germantown, MD, USA). Briefly, 2 µg of plasmid pStx2 or pGEM-T religated vector were mixed with 15 µL of Polyfect reagent following the manufacturer’s instructions. After 10 min, the cells were incubated with the transfection mix (DNA-polyfect) using complete medium at 37 °C in 5% CO_2_. Supernatants were collected and cell monolayer were lysed in TRIzol for total RNA isolation 18 h later.

### 4.11. Quantitative RT-qPCR

For total RNA isolation, macrophages HCT-8 or 293T cells were lysed in TRIzol according to the manufacturer’s instructions. For all RNA samples, 1µg was treated with RQ1 RNase-free DNase (Promega Inc., Madison, WI, USA). Then cDNA was synthesized using Superscript III reverse transcriptase (Thermo Fisher Scientific, Waltham, MA, USA) and gene-specific primer (*stx_2_*-A 5′-ACACAGGAGCAGTTTCAGACAG-3′ and *stx_2_*-B 5′-GAATTCTCAGTCATTATTAAACTG-3′), random hexamers (Biodynamics SRL, Buenos Aires, Argentina) or oligo (dT) primers (Biodynamics SRL, Buenos Aires, Argentina) according to the manufacturer’s guidelines (Invitrogen Life Technologies, San Diego, CA, USA). cDNAs were amplified using Taq DNA polymerase (Invitrogen Life Technologies, San Diego, CA, USA) with SYBR green using an Eppendorf Mastercycler. Primers and PCR conditions for the *stx_2_-A* subunit, *stx_2_-B* subunit, and *SDHA* are listed in Table 1andTable 2, respectively.

Data was analyzed with Realplex software using the relative standard curve method. Reactions were performed in duplicates or triplicates.

### 4.12. Statistical Analysis

Data are expressed as mean ± Standard Error of the Mean (SEM) and were analyzed by t-test, one-way ANOVA, or two-way ANOVA as indicated in legend, always using Tukey’s Multiple comparisons post-test. Data were analyzed using Graphpad Prism 8. *p* values less than 0.05 were considered statistically significant.

## Figures and Tables

**Table 1:** Sequences.

Sequence	Forward (5′–3′)	Reverse (5′–3′)
*stx_2_-A* subunit	TGGCGTTAATGGAGTTCAGTGG	ACACAGGAGCAGTTTCAGACAG
*stx_2_-B* subunit	AGTCGCTGGAATCTGCAACCGTTAC	TCAGCAAATCCGGAGCCTGATTCAC
*SDHA*	AAGTCCCTCCAATTAAACCAAACG	GTCTTCAGGTGCTTTAGGTCTCC

**Table 2:** Conditions.

Sequence	[Mg^2+^](mM)	PCR Program
*stx_2_-A* subunit	2.5	95 °C × 2 min, 40 cycles of: [95 °C × 15 sec, 60 °C × 20 sec, 68 °C × 20 sec]
*stx_2_-B* subunit	2.5	95 °C × 2 min, 40 cycles of: [95 °C × 15 sec, 60 °C × 20 sec, 68 °C × 20 sec]
*SDHA*	4	95 °C × 2 min, 40 cycles of: [95 °C × 15 sec, 60 °C × 20 sec, 68 °C × 20 sec]

## Supplementary Materials

The following are available online at https://www.mdpi.com/2072-6651/12/1/48/s1, Figure S1: Stx2 activity in lysates from infected macrophages and HCT-8 cells.
