# Supplementary material for: Role of Shiga Toxins in Cytotoxicity and Immunomodulatory Effects of Escherichia coli O157:H7 during Host-Bacterial Interactions in vitro
Source: Toxins (Basel). 2020 Jan 14;12(1):48. doi: 10.3390/toxins12010048 (PMC7020462; doi:10.3390/toxins12010048)
Supplement: Supplementary file 1 [file toxins-12-00048-s001.pdf]

Supplementary Materials: Role of Shiga Toxins in Cytotoxicity and Immunomodulatory Effects of *Escherichia coli* O157:H7 during Host-Bacterial Interactions in vitro

Andrea Cecilia Bruballa, Carolina Maiumi Shiromizu, Alan Mauro Bernal, Gonzalo Ezequiel Pineda, Florencia Sabbione, Analia Silvina Trevani, Leticia Verónica Bentancor, María Victoria Ramos, Romina Jimena Fernández-Brando, Manuel Javier Muñoz and Marina Sandra Palermo *

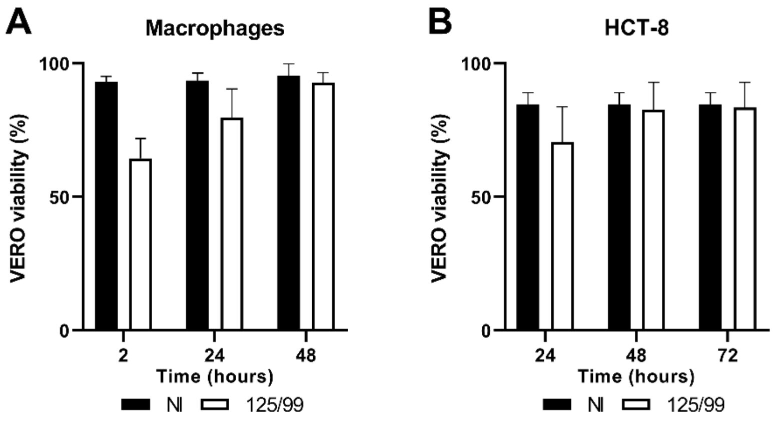

Figure S1. Stx2 activity in lysates from infected macrophages and HCT-8 cells. Lysates from macrophages (**A**) or HCT-8 cells (**B**) were obtained at the indicated times after infection with 125/99. Lysates of non-infected cells were used as controls (NI) and all values were normalized considering Vero cells cultured in medium 1:2 PBS without any lysates 100% viability. Data represent mean ± SEM for biological replicates (n = 3).
